# Guidelines for the Treatment of Latent Tuberculosis Infection: Recommendations from the National Tuberculosis Controllers Association and CDC, 2020

**DOI:** 10.15585/mmwr.rr6901a1

**Published:** 2020-02-14

**Authors:** Timothy R. Sterling, Gibril Njie, Dominik Zenner, David L. Cohn, Randall Reves, Amina Ahmed, Dick Menzies, C. Robert Horsburgh, Charles M. Crane, Marcos Burgos, Philip LoBue, Carla A. Winston, Robert Belknap

**Affiliations:** ^1^Vanderbilt University Medical Center, Nashville, Tennessee; ^2^National Center for HIV/AIDS, Viral Hepatitis, STD, and TB Prevention, Division of Tuberculosis Elimination, CDC, Atlanta, Georgia; ^3^Institute for Global Health, University College London, London, England; ^4^Denver Health and Hospital Authority, Denver, Colorado; ^5^Levine Children’s Hospital, Charlotte, North Carolina; ^6^Montreal Chest Institute and McGill International TB Centre, Montreal, Canada; ^7^Boston University Schools of Public Health and Medicine, Boston, Massachusetts; ^8^National Tuberculosis Controllers Association, Smyrna, Georgia; ^9^University of New Mexico Health Science Center and New Mexico Department of Health, Albuquerque, New Mexico

## Abstract

Comprehensive guidelines for treatment of latent tuberculosis infection (LTBI) among persons living in the United States were last published in 2000 (American Thoracic Society. CDC targeted tuberculin testing and treatment of latent tuberculosis infection. Am J Respir Crit Care Med 2000;161:S221–47). Since then, several new regimens have been evaluated in clinical trials. To update previous guidelines, the National Tuberculosis Controllers Association (NTCA) and CDC convened a committee to conduct a systematic literature review and make new recommendations for the most effective and least toxic regimens for treatment of LTBI among persons who live in the United States.

The systematic literature review included clinical trials of regimens to treat LTBI. Quality of evidence (high, moderate, low, or very low) from clinical trial comparisons was appraised using the Grading of Recommendations Assessment, Development, and Evaluation (GRADE) criteria. In addition, a network meta-analysis evaluated regimens that had not been compared directly in clinical trials. The effectiveness outcome was tuberculosis disease; the toxicity outcome was hepatotoxicity. Strong GRADE recommendations required at least moderate evidence of effectiveness and that the desirable consequences outweighed the undesirable consequences in the majority of patients. Conditional GRADE recommendations were made when determination of whether desirable consequences outweighed undesirable consequences was uncertain (e.g., with low-quality evidence).

These updated 2020 LTBI treatment guidelines include the NTCA- and CDC-recommended treatment regimens that comprise three preferred rifamycin-based regimens and two alternative monotherapy regimens with daily isoniazid. All recommended treatment regimens are intended for persons infected with Mycobacterium tuberculosis that is presumed to be susceptible to isoniazid or rifampin. These updated guidelines do not apply when evidence is available that the infecting M. tuberculosis strain is resistant to both isoniazid and rifampin; recommendations for treating contacts exposed to multidrug-resistant tuberculosis were published in 2019 (Nahid P, Mase SR Migliori GB, et al. Treatment of drug-resistant tuberculosis. An official ATS/CDC/ERS/IDSA clinical practice guideline. Am J Respir Crit Care Med 2019;200:e93–e142). The three rifamycin-based preferred regimens are 3 months of once-weekly isoniazid plus rifapentine, 4 months of daily rifampin, or 3 months of daily isoniazid plus rifampin. Prescribing providers or pharmacists who are unfamiliar with rifampin and rifapentine might confuse the two drugs. They are not interchangeable, and caution should be taken to ensure that patients receive the correct medication for the intended regimen. Preference for these rifamycin-based regimens was made on the basis of effectiveness, safety, and high treatment completion rates. The two alternative treatment regimens are daily isoniazid for 6 or 9 months; isoniazid monotherapy is efficacious but has higher toxicity risk and lower treatment completion rates than shorter rifamycin-based regimens.

In summary, short-course (3- to 4-month) rifamycin-based treatment regimens are preferred over longer-course (6–9 month) isoniazid monotherapy for treatment of LTBI. These updated guidelines can be used by clinicians, public health officials, policymakers, health care organizations, and other state and local stakeholders who might need to adapt them to fit individual clinical circumstances.

## Introduction

One fourth of the global population (approximately 2 billion persons) is estimated to be infected with *Mycobacterium tuberculosis* (*1*), including approximately 13 million in the United States (*2*). Most infected persons are asymptomatic and classified as having latent tuberculosis infection (LTBI). If untreated, approximately 5%–10% of persons with LTBI progress to tuberculosis (TB) disease during their lifetime (*3*–*5*). Progression from untreated LTBI accounts for approximately 80% of U.S. TB disease cases (*6*). Treatment of LTBI is effective in preventing progression to TB disease (*7*). The most recent comprehensive guidelines for treatment of LTBI in the United States were published in 2000 (*8*). In 2003, CDC and the American Thoracic Society recommended against use of the 2-month regimen of rifampin plus pyrazinamide because of the risk for severe hepatotoxicity (*9*). Since then, several new regimens have been evaluated in clinical trials. To update the 2000 and 2003 treatment guidelines, the National Tuberculosis Controllers Association (NTCA) and CDC convened a committee to conduct a systematic literature review of clinical trials for the treatment of LTBI. Grading of Recommendations Assessment, Development, and Evaluation (GRADE) criteria were applied to the evidence of effectiveness, a network meta-analysis of selected evidence was performed, and the evidence was used to support 2020 LTBI treatment guidelines.

These updated 2020 LTBI treatment guidelines apply to persons with LTBI who live in the United States. In addition, these guidelines apply to persons infected with *M. tuberculosis* that is presumed to be susceptible to isoniazid or rifampin; they do not apply when evidence is available that the infecting *M. tuberculosis* strain is resistant to both isoniazid and rifampin. Local and state TB programs in the United States answer questions about diagnosing and treating persons with LTBI in their jurisdictions (http://www.tbcontrollers.org).­

## Methods

These updated guidelines were developed by NTCA and CDC. The LTBI treatment guidelines committee members, who are the authors of this report, were nominated on the basis of their expertise in treatment of LTBI. The committee had expertise in epidemiology, domestic and international TB control, clinical trials, and treatment of LTBI in adults and children. A methodologist with expertise in the GRADE approach served as a consultant to the guideline development committee.

### Evidence Search

The committee determined that the following clinical question should be addressed in the updated guidelines: “Which regimens for treatment of latent tuberculosis infection have the greatest effectiveness and least toxicity?” The question was written in the population, intervention, comparator, outcomes (PICO) format, and then the outcomes were rated as critical, important, or not important. Comparison of regimen toxicities was limited to hepatotoxicity because this was the only toxicity that could be consistently compared across studies.

A systematic literature review was initiated in December 2017. Electronic databases including MEDLINE, Embase, CINAHL, ClinicalTrials.gov, the Cochrane Central Register of Controlled Trials (CENTRAL), and gray literature were searched for studies evaluating the effectiveness of LTBI treatment regimens. Search terms included “latent tuberculosis,” “latent TB,” “LTBI,” “*Mycobacterium tuberculosis,*” “tuberculosis infection” AND “isoniazid,” “rifampin,” “rifapentine,” or “pyrazinamide.” Articles were included if the study design was a randomized controlled trial and outcomes included prevention of TB disease and drug-related hepatotoxicity. Studies that included persons with suspected or confirmed TB disease were excluded from the review.

The initial search located a high-quality systematic review and meta-analysis published in August 2017 that examined the effectiveness of LTBI treatment regimens (*10*). The study authors were contacted and asked for access to the extracted data. Study characteristics, types of participants, interventions, the outcomes measured, and results were extracted from each study. If the data were amenable to pooling, effects were estimated via meta-analysis. For the meta-analyses, a random effects model was used unless otherwise specified, and effect estimates were reported as odds ratios. All statistical analyses were conducted using the “metafor” package in R, versions 3.4.3 (*11*). The Cochrane risk-of-bias tool was used to conduct a bias assessment (*12*). Analyses conducted in 2018 included combined data from the studies in the previous review and articles identified during an updated search for studies published during June 2017–August 2018 (Figure) (*13*,*14*).

**FIGURE:**
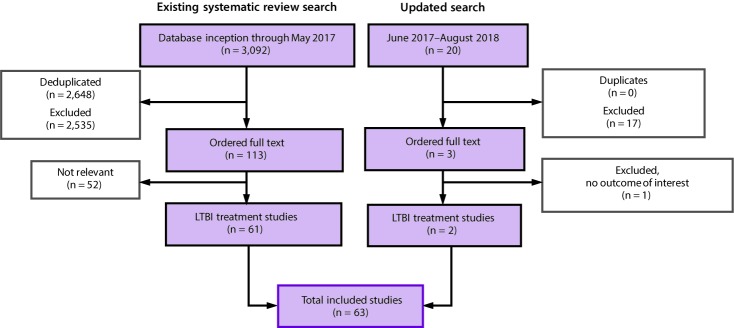
Systematic literature review search process* for latent tuberculosis infection treatment regimens recommended by the National Tuberculosis Controllers Association and CDC, 2020 **Abbreviation:** LTBI = latent tuberculosis infection. * Existing systematic review search: the results from the 2017 analysis were published, citing all primary studies included in the analysis (Zenner D, Beer N, Harris RJ, Lipman MC, Stagg HR, van der Werf MJ. Treatment of latent tuberculosis infection: an updated network meta-analysis. Ann Intern Med 2017;167:248–5). Updated search: analyses included combined data from the studies included in the previous review and articles identified during an updated search for studies published during June 2017–August 2018.

All treatment regimens were analyzed using a Bayesian network meta-analysis (NMA) approach, which allowed for indirect comparisons of treatment regimens when direct comparisons were not available. However, direct, pairwise meta-analysis was the preferred method; the results of the network analysis are presented in this report only if no direct comparisons were available. A full description of the network analysis method has been previously published (*10*,*15*). NMA allows for indirect comparisons of treatment regimens through inference from a network of evidence. For this analysis, WinBUGS software (version 1.4; Medical Research Council Biostatistics Unit of the University of Cambridge) was used to create the Bayesian network with posterior distributions on the basis of 20,000 samples after a burn-in period of 10,000 iterations (*15*). Convergence was assessed by inspecting parameter chains and the Gelman–Rubin diagnostic (*16*). Summary statistics and 95% credible intervals were obtained from posterior distributions. Network inconsistency, which can arise if indirect comparisons conflict with direct pairwise estimates, was assessed by comparison with standard meta-analysis and by using the omnibus test for consistency (*17*).

The overall quality of evidence was appraised using the GRADE approach, and GRADEpro software was used to develop evidence profiles that summarized the quality of evidence for each outcome (high, moderate, low, or very low) and the rationale for the quality of evidence appraisal (*18*). Head-to-head comparisons of regimens evaluated in clinical trials were evaluated according to the populations studied: adults, children, HIV positive, and HIV negative. References for all of the studies included in the analyses are available (Supplementary Tables; https://stacks.cdc.gov/view/cdc/84235).

### Development of Recommendations

The committee discussed evidence during face-to-face meetings and teleconferences. GRADE evidence tables were prioritized according to the regimens, comparisons, and study populations that were deemed most clinically relevant to the United States. If discrepancies between GRADE head-to-head comparisons and network meta-analysis results were found, the committee prioritized the GRADE comparisons. Recommendations were formulated on the basis of the following considerations: the balance of desirable consequences of the intervention (benefits) and undesirable consequences (regimen complexity, adverse effects, and cost), the quality of evidence, patient values and preferences, and feasibility (*19*). The desirable and undesirable consequences considered by the committee included both those related to individuals and to overall public health.

A strong GRADE recommendation for a regimen was made if the panel concluded that the desirable consequences of the intervention outweighed the undesirable consequences, the majority of well-informed patients would choose the regimen, and the evidence was at least moderate quality (*18*,*19*). A conditional GRADE recommendation was made for a regimen when uncertainty existed regarding whether the desirable consequences outweighed the undesirable consequences (e.g., low-quality evidence for a critical outcome such that additional evidence could change key findings, hence the recommendation) (*18*,*19*). A conditional recommendation indicates that well-informed patients might make different choices regarding whether to choose the regimen (*18*,*19*).

The panel also prioritized recommended regimens as either preferred or alternative. Preferred regimens were defined as having excellent tolerability and efficacy, shorter treatment duration, and higher completion rates. Alternative regimens were defined as having excellent efficacy but longer treatment duration and lower completion rates. The rationale for prioritizing the regimens was that treatment completion rates are higher with shorter regimens (*20*); if regimens have similar efficacy and safety, the shorter regimen is more effective because completion rates are higher.

Draft recommendations were publicly presented during the U.S. Advisory Council on the Elimination of Tuberculosis meeting on December 11, 2018, and at the NTCA meeting on April 23, 2019. The recommendations were positively received at both meetings, and no substantive changes were made to the recommendations thereafter.

## Results

The GRADE evidence tables are provided (Table 1) (Supplementary Tables; https://stacks.cdc.gov/view/cdc/84235). The Supplementary Tables contain all references; selected references are included in this report. In total, 55 clinical trials evaluated effectiveness (*7*,*13*,*14*,*21*–*74*), and 31 trials evaluated toxicity (*13*,*14*,*27*,*35*–*38*,*43*–*46*,*49*,*51*–*53*,*55*,*61*–*66*,*68*,*71*,*72*,*75*–*82*). Results of the 2018 updated network meta-analysis are provided (Table 2); 63 studies of 16 regimens were evaluated (*7*,*13*,*14*,*21*–*82*).

**TABLE 1:** Summary of GRADE evidence tables, by treatment regimen and study population*

Regimen		Population	No. of trials	
Experimental regimen	Comparator regimen		Effectiveness	Toxicity
3 mos isoniazid plus rifapentine given once weekly	9 mos isoniazid	HIV-positive adults	1	1
3 mos isoniazid plus rifapentine given once weekly	9 mos isoniazid	HIV-negative adults and children	1	1
3 mos isoniazid plus rifapentine given once weekly	9 mos isoniazid	HIV-negative children	1	1
3 mos isoniazid plus rifapentine given once weekly	6 mos isoniazid	HIV-positive adults	1	1
3 mos isoniazid plus rifampin given daily	9 mos isoniazid	HIV-negative adults	1	1
3 mos isoniazid plus rifampin given daily	6 mos isoniazid	HIV negative adults and children	3	2
3 mos isoniazid plus rifampin given daily	6 mos isoniazid	HIV-positive adults	4	4
3 mos isoniazid plus rifampin given daily	Placebo or no treatment	HIV-positive adults	2	1
3 mos isoniazid plus rifampin given daily	Placebo or no treatment	HIV-negative adults and children	2	0
4 mos rifampin given daily	9 mos isoniazid	HIV-negative adults	1	2
4 mos rifampin given daily	9 mos isoniazid	HIV-negative children	1	1
4 mos rifampin given daily	6 mos isoniazid	HIV-negative children	1	0
6 mos isoniazid given daily	Placebo	HIV-negative adults and children	4	2
6 mos isoniazid given daily	Placebo or no treatment	HIV-positive adults	5	3
9 mos isoniazid given daily	No treatment	HIV-negative adults and children	2	0
12 mos isoniazid given daily	No treatment	HIV-positive adults	2	0
12 mos isoniazid given daily	Placebo	HIV-positive adults and children	5	3
12 mos isoniazid given daily	Placebo	HIV-positive children	3	1
12 mos isoniazid given daily	Placebo or no treatment	HIV-negative adults and children	15	5
3 mos isoniazid plus rifapentine given once weekly	Continuous isoniazid (up to 6 yrs)	HIV-positive adults	1	1
2 mos rifampin and pyrazinamide given daily or twice weekly	6 mos isoniazid, 12 mos isoniazid	HIV-positive adults and children	4	2

**Abbreviation:** GRADE = Grading of Recommendations Assessment, Development, and Evaluation.

* Study details and information on evidence quality are available (Supplementary Tables; https://stacks.cdc.gov/view/cdc/84235).

**TABLE 2:** Network meta-analysis of regimens to treat latent tuberculosis infection

Risk and treatment	2017*	2018 update (unpublished)
	Odds ratio (95% credible interval)	Odds ratio (95% credible interval)
**Tuberculosis risk compared with no treatment**		
No treatment	1 (ref)	1 (ref)
3 mos isoniazid plus rifapentine given once weekly	0.36 (0.18–0.73)	0.36 (0.18–0.72)
3–4 mos rifampin given daily	0.25 (0.11–0.57)	0.25 (0.12–0.50)
3 mos isoniazid plus rifampin given daily	0.33 (0.20–0.54)	0.33 (0.20–0.53)
6 mos isoniazid given daily	0.40 (0.26–0.60)	0.40 (0.26–0.59)
9 mos isoniazid given daily	0.46 (0.22–0.95)	0.47 (0.24–0.90)
**Hepatotoxicity risk compared with no treatment**		
No treatment	1 (ref)	1 (ref)
3 mos isoniazid plus rifapentine given once weekly	0.52 (0.13–2.15)	0.53 (0.13–2.13)
3–4 mos rifampin given daily	0.14 (0.02–0.81)	0.13 (<0.02–0.72)
3 mos isoniazid plus rifampin given daily	0.72 (0.21–2.37)	0.73 (0.22–2.38)
6 mos isoniazid given daily	1.10 (0.40–3.17)	1.11 (0.41–3.15)
9 mos isoniazid given daily	1.70 (0.35–8.05)	1.77 (0.35–8.32)

**Abbreviation:** ref = referent.

* The results from the 2017 analysis were published, citing all primary studies included in the analysis (Zenner D, Beer N, Harris RJ, Lipman MC, Stagg HR, van der Werf MJ. Treatment of latent tuberculosis infection: an updated network meta-analysis. Ann Intern Med 2017;167:248–55.); the 2018 update includes data subsequently published (Diallo T, Adjobimey M, Ruslami R, et al. Safety and side effects of rifampin versus isoniazid in children. N Engl J Med 2018;379:454–63; Menzies D, Adjobimey M, Ruslami R, et al. Four months of rifampin or nine months of isoniazid for latent tuberculosis in adults. N Engl J Med 2018;379:440–53).

### Summary of Evidence and Recommendations

The recommended treatment regimens include three preferred and two alternative treatment regimens (Tables 3 and 4). Rifamycin-based regimens, including 3 months of once-weekly isoniazid plus rifapentine, 4 months of daily rifampin, and 3 months of daily isoniazid plus rifampin are the preferred recommended regimens because of their effectiveness, safety, and high treatment completion rates. Regimens of 6 or 9 months of daily isoniazid are alternative recommended regimens; although efficacious, they have higher toxicity risk and lower treatment completion rates, which decrease effectiveness. On the basis of the most recent comprehensive LTBI treatment guidelines in the United States, which were published in 2000 (*8*), 9 months of daily isoniazid was considered the standard comparator regimen to evaluate shorter-course regimens. Data on the effectiveness and toxicity of 9 months of daily isoniazid are provided, as are data on the other recommended regimens. A rifamycin-based regimen refers to treatment that includes either rifampin or rifapentine. 

**TABLE 3:** Recommendations for regimens to treat latent tuberculosis infection

Priority rank*	Regimen	Recommendation (strong or conditional)	Evidence (high, moderate, low, or very low)
Preferred	3 mos isoniazid plus rifapentine given once weekly	Strong	Moderate
Preferred	4 mos rifampin given daily	Strong	Moderate (HIV negative)^†^
Preferred	3 mos isoniazid plus rifampin given daily	Conditional	Very low (HIV negative)
		Conditional	Low (HIV positive)
Alternative	6 mos isoniazid given daily	Strong^§^	Moderate (HIV negative)
		Conditional	Moderate (HIV positive)
Alternative	9 mos isoniazid given daily	Conditional	Moderate

**Abbreviation:** HIV = human immunodeficiency virus.

* *Preferred:* excellent tolerability and efficacy, shorter treatment duration, higher completion rates than longer regimens and therefore higher effectiveness; *alternative:* excellent efficacy but concerns regarding longer treatment duration, lower completion rates, and therefore lower effectiveness.

^†^ No evidence reported in HIV-positive persons.

^§^ Strong recommendation for those persons unable to take a preferred regimen (e.g., due to drug intolerability or drug-drug interactions).

**TABLE 4:** Dosages for recommended latent tuberculosis infection treatment regimens

Drug	Duration	Dose and age group	Frequency	Total doses
Isoniazid* and rifapentine^†^	3 mos	**Adults and children aged ≥12 yrs**	Once weekly	12
		Isoniazid: 15 mg/kg rounded up to the nearest 50 or 100 mg; 900 mg maximum		
		Rifapentine:		
		10–14.0 kg, 300 mg		
		14.1–25.0 kg, 450 mg		
		25.1–32.0 kg, 600 mg		
		32.1–49.9 kg, 750 mg		
		≥50.0 kg, 900 mg maximum		
		**Children aged 2–11 yrs**		
		Isoniazid*: 25 mg/kg; 900 mg maximum		
		Rifapentine^†^: see above		
Rifampin^¶^	4 mos	**Adults:** 10 mg/kg	Daily	120
		**Children:** 15–20 mg/kg**		
		**Maximum dose:** 600 mg		
Isoniazid* and rifampin^¶^	3 mos	**Adults**	Daily	90
		Isoniazid*: 5 mg/kg; 300 mg maximum		
		Rifampin^¶^: 10 mg/kg; 600 mg maximum		
		**Children**		
		Isoniazid*: 10–20 mg/kg^††^; 300 mg maximum		
		Rifampin^¶^: 15–20 mg/kg; 600 mg maximum		
Isoniazid*	6 mos	**Adults:** 5 mg/kg	Daily	180
		**Children:** 10–20 mg/kg^††^		
		**Maximum dose:** 300 mg		
		**Adults:**15 mg/kg	Twice weekly^§^	52
		**Children:** 20–40 mg/kg^††^		
		**Maximum dose:** 900 mg		
	9 mos	**Adults:** 5 mg/kg	Daily	270
		**Children:** 10–20 mg/kg^††^		
		**Maximum dose:** 300 mg		
		**Adults:** 15 mg/kg	Twice weekly^§^	76
		**Children:** 20–40 mg/kg^††^		
		**Maximum dose:** 900 mg		

* Isoniazid is formulated as 100-mg and 300-mg tablets. ^†^ Rifapentine is formulated as 150-mg tablets in blister packs that should be kept sealed until use. ^§^ Intermittent regimens must be provided via directly observed therapy (i.e., a health care worker observes the ingestion of medication). ^¶^ Rifampin (rifampicin) is formulated as 150-mg and 300-mg capsules. ** The American Academy of Pediatrics acknowledges that some experts use rifampin at 20–30 mg/kg for the daily regimen when prescribing for infants and toddlers (**Source:** American Academy of Pediatrics. Tuberculosis. In: Kimberlin DW, Brady MT, Jackson MA, Long SS, eds. Red Book: 2018 Report of the Committee on Infectious Diseases. 31st ed. Itasca, IL: American Academy of Pediatrics; 2018:829–53). ^††^ The American Academy of Pediatrics recommends an isoniazid dosage of 10–15 mg/kg for the daily regimen and 20–30 mg/kg for the twice-weekly regimen.

#### Preferred Regimens

##### Three Months of Weekly Isoniazid Plus Rifapentine 

A regimen of 3 months of once-weekly isoniazid plus rifapentine is a preferred regimen that is strongly recommended for adults and children aged >2 years, including HIV-positive persons (as drug interactions allow). This regimen, administered through directly observed therapy, had equivalent effectiveness and was not more toxic than the standard regimen of 9 months of daily isoniazid in adults and children aged >2 years (*53*,*68*,*83*). Treatment completion rates were higher with the 3-month regimen. In HIV-negative persons in a noninferiority study, 3 months of isoniazid and rifapentine was equivalent to and was associated with less hepatoxicity than 9 months of isoniazid, despite more discontinuation because of adverse effects (*68*). In HIV-positive persons, no significant difference was found in a comparison of isoniazid plus rifapentine for all outcomes with either 6 or 9 months of isoniazid (*22*,*53*). In a noninferiority study of 3 months of weekly isoniazid plus rifapentine, the completion rate by self-administered therapy was inferior to the rate with direct observation but noninferior in the prespecified subpopulation from the United States (*84*).

Potential disadvantages of this regimen include cost of medications that are greater than most alternatives, potential added costs if provided by directly observed therapy (with treatment completion being highest with directly observed therapy, although self-administered therapy is an approved option) (*85*), the need to take numerous pills simultaneously (10 pills once weekly compared with two or three pills daily for other regimens for most adults), and the association with a systemic drug reaction or influenza-like syndrome that can include syncope and hypotension. Severe events requiring hospitalization occurred in 0.1% of persons (*68*,*86*). The systemic drug reaction is self-limited and usually mild; no deaths have been reported. Potential drug interactions and acquired drug resistance if TB disease is not adequately excluded also are important considerations for all treatment regimens.

##### Four Months of Daily Rifampin

A regimen of 4 months of daily rifampin is a preferred treatment that is strongly recommended for HIV-negative adults and children of all ages. (No evidence is available for effectiveness in HIV-positive persons.) The effectiveness of this regimen was clinically equivalent to, and less toxic than, the standard regimen of 9 months of daily isoniazid in adults and children (*13*,*14*,*78*,*79*). Four months of daily rifampin had noninferior effectiveness in preventing TB disease compared with 9 months of daily isoniazid, as well as a lower rate of treatment discontinuation because of adverse effects, a lower rate of hepatotoxicity, and a higher rate of treatment completion (*13*,*14*).

The potential disadvantages of the rifamycin-based regimens are the many drug interactions, including warfarin, oral contraceptives, azole antifungals, and HIV antiretroviral therapy (*87*). Rifabutin has fewer or less pronounced drug interactions and may be used in place of rifampin when rifampin is contraindicated due to drug-drug interactions and isoniazid cannot be used (*87*). Drug interactions with weekly rifapentine are fewer than with rifampin and appear to be fewer than with rifabutin; therefore, weekly isoniazid and rifapentine could be considered when rifampin is contraindicated, although clinical data are limited (*88*). Drug-drug interactions between rifamycins and antiretroviral therapy are regularly updated by the U.S. Department of Health and Human Services (https://aidsinfo.nih.gov/guidelines/html/4/adult-and-adolescent-opportunistic-infection/0). In HIV-positive persons with low CD4+ lymphocyte counts, the risk for asymptomatic or subclinical TB disease increases, possibly facilitating rifampin resistance if TB disease is inadvertently treated with rifampin monotherapy (*89*).

##### Three Months of Daily Isoniazid Plus Rifampin

A regimen of 3 months of daily isoniazid plus rifampin is a preferred treatment that is conditionally recommended for adults and children of all ages and for HIV-positive persons as drug interactions allow. HIV-negative adults and children with a positive tuberculin skin test (TST) who received 3 months of daily isoniazid plus rifampin appeared to have a similar risk for TB disease, hepatotoxicity, and adverse effects requiring discontinuation of therapy as those who received ≥6 months of isoniazid (*23*,*35*,*44*,*51*,*90*). Among children aged <15 years specifically, a 3-month course of daily isoniazid plus rifampin appeared as effective as a 6-month or longer course of isoniazid, because direct comparisons found no difference in TB disease and no differences in adverse effects requiring discontinuation of therapy or hepatotoxicity (*67*). In HIV-positive persons, no difference was found in the incidence of TB disease among those who received 3 months of daily isoniazid plus rifampin compared with those who received ≥6 months of isoniazid monotherapy, regardless of whether they were TST positive, TST negative, or anergic (*34*,*46*,*63*,*72*). Hepatotoxicity was less frequent among those receiving the shorter course of therapy, although discontinuation of therapy because of adverse effects was more frequent (*63*).

Potential drug interactions with rifampin and acquired drug resistance if TB disease is not adequately excluded also are important considerations (see previous section on 4 months of daily rifampin). In addition, hepatotoxicity risk might be greater with the two drugs given together than with either drug given alone (*91*).

#### Alternative Regimens: Six or Nine Months of Daily Isoniazid

Regimens of 6 or 9 months of daily isoniazid are alternative recommended regimens; 6 months daily is strongly recommended for HIV-negative adults and children of all ages and conditionally for HIV-positive adults and children of all ages and 9 months daily is conditionally recommended for adults and children of all ages, both HIV-negative and HIV-positive. Isoniazid reduces the risk for developing TB disease in persons with a positive TST, including HIV-negative adults and children (*7*,*23*,*28*,*43*,*47*,*73*), HIV-positive adults (*27*,*38*,*42*,*46*,*60*,*72*), and presumably also HIV-positive children. The drug can cause hepatotoxicity and be associated with discontinuation because of adverse effects, although these effects are more common in adults than children (*23*,*43*).

In HIV-positive persons who have a negative TST, anergy, or an unknown TST, the benefit of isoniazid is uncertain in settings with low TB incidence (*38*). For these HIV-positive persons, the potential exists for a reduction in the incidence of TB disease and an increase in adverse effects with isoniazid therapy; however, the likelihood of these effects remains uncertain because of wide confidence intervals resulting from too few events.

The evidence synthesis included multiple durations of isoniazid therapy in persons with a positive TST (3, 6, and 12 months in HIV-negative persons and 6 months in HIV-positive persons) (*7*,*72*). Among HIV-negative persons with inactive TB (defined as the presence of tuberculin positivity, stable fibrotic lung lesions, and negative sputum cultures in persons not previously treated), 6 and 12 months of therapy were more effective than 3 months of therapy, demonstrating the benefit of LTBI treatment with isoniazid in this high-risk subset of patients with LTBI (*7*). Studies of other regimens have persons with LTBI and fibrotic lesions but in much smaller numbers (*14*,*68*). According to the results of the systematic review process, among HIV-positive persons, 6 months of therapy was highly effective (*72*), and the effect of other durations was unknown. Also reviewed was an analysis that included different, fewer trials than included in this report and found that 9 months of daily isoniazid therapy was perhaps more effective than 6 months and similar to 12 months (*25*,*92*–*94*). However, no clinical trial data were available directly comparing 9 months of isoniazid to placebo, 6 months of isoniazid, or 12 months of isoniazid.

Among HIV-positive persons living in areas with a high TB incidence, isoniazid is complementary to antiretroviral therapy in preventing TB disease. Two randomized controlled trials have demonstrated that isoniazid plus antiretroviral therapy decreased the incidence of TB disease to a greater extent than either isoniazid alone or antiretroviral therapy alone (*27*,*61*). Potential disadvantages of the regimen include its long duration, hepatoxicity, and low treatment completion rates (primarily due to the first two factors).

## Discussion

A systematic literature review was performed of clinical trial data pertaining to effectiveness and toxicity of treatment of LTBI, including studies published since the 2018 World Health Organization LTBI guidelines (*95*). Evidence quality was evaluated using the GRADE approach, and a network meta-analysis was performed, updated to include data from studies published since a previous network meta-analysis (*10*), to compare regimens not evaluated head-to-head in clinical trials. 

Recommendations were formulated on the basis of the balance of desirable and undesirable consequences of the intervention, the quality of evidence, patient values and preferences, and feasibility. These factors also informed the priority rank of the regimens as preferred or alternative, with preference for shorter regimens, given their similar efficacy compared with 6–9 months of isoniazid but favorable tolerability and higher treatment completion rates. This combination of characteristics should result in greater effectiveness of the shorter regimens in clinical settings. More effective treatment of LTBI will facilitate TB elimination (*96*). Prescribing providers or pharmacists who are unfamiliar with rifampin and rifapentine might confuse the two drugs. They are not interchangeable, and caution should be taken to ensure that patients receive the correct medication for the intended regimen.

Although 9 months of isoniazid was a preferred regimen in the guidelines published in 2000, both 6 and 9 months of isoniazid were recommended at that time (*8*). In these current guidelines, application of GRADE criteria resulted in a strong recommendation for 6 months of isoniazid as an alternative for those persons unable to take a shorter preferred regimen (e.g., due to drug intolerability or drug-drug interactions), particularly in HIV-negative persons. The longer duration of isoniazid could increase the risk for hepatotoxicity and although increased effectiveness is plausible, the two treatment durations have not been directly compared.

Two months of rifampin plus pyrazinamide are not recommended for treatment of LTBI because of the hepatotoxicity risk. However, in persons treated empirically for TB disease with isoniazid, rifampin, and pyrazinamide for 2 months, this regimen will effectively treat LTBI in persons subsequently determined to have LTBI rather than TB disease.

## Other Considerations

Following are several considerations for the use of these guidelines. First, the committee did not include cost-effectiveness in evaluating the evidence; recommendations were based on evaluating effectiveness and toxicity of the regimens. Second, the committee did not evaluate evidence regarding how to implement these regimens programmatically (e.g., who to test and treat and management of side effects). Third, these guidelines focus on treatment regimens for persons with LTBI living in countries with low TB disease incidence. These guidelines do not address other empiric TB prevention strategies (e.g., 1 month of isoniazid plus rifapentine among HIV-positive persons living in settings with a high TB incidence regardless of results from the TST or an interferon-gamma release assay) (*97*). Finally, shorter regimens should not be used for patients in whom rifamycins are contraindicated, including those taking medications with significant drug-drug interactions with rifamycins.

## Conclusion

For patients without drug intolerability or drug-drug interactions, short-course (3–4 months) rifamycin-based treatment regimens are preferred over the longer-course (6–9 months) isoniazid monotherapy for treatment of LTBI. These guidelines can be used by clinicians, public health officials, policymakers, health care organizations, and other state and local stakeholders who might need to adapt these guidelines for individual clinical circumstances. Local and state TB programs in the United States answer questions about diagnosing and treating persons with LTBI in their jurisdictions (http://www.tbcontrollers.org).
